# Examining the Genomic Influence of Skin Antioxidants *In Vitro*

**DOI:** 10.1155/2010/230450

**Published:** 2010-07-12

**Authors:** James V. Gruber, Robert Holtz

**Affiliations:** ^1^Arch Personal Care, 70 Tyler Place, South Plainfield, NJ 07080, USA; ^2^Bioinnovation Laboratory, 7220 W. Jefferson Avenue, STE 112, Lakewood, CO 80235, USA

## Abstract

A series of well-known, purified antioxidants including: Resveratrol, Epigallocatechin Gallate (EGCG), Genistein, Rosavin, Puerarin, Chlorogenic Acid, Propolis and two newer unexplored isoflavonoids isolated from *Maclura pomifera* (Osage Orange) including Pomiferin and Osajin, were applied to Normal Human Dermal Fibroblasts (NHDF) and Normal Human Dermal Keratinocytes (NHEK) for 24 hours. The resulting treated cells were then examined using human gene microarrays supplied by Agilent. These chips typically have somewhere on the order of 30,000 individual genes which are expressed in the human genome. For our study, this large list of genes was reduced to 205 principal genes thought to be important for skin and each individual ingredient was examined for its influence on the culled list of genes. Working on a hypothesis that there may be some common genes which are either upregulated or downregulated by all or most of these ingredients, a short list of genes for each cell line was developed. What appears to emerge from these studies is that several genes in the gene pool that was screened are influenced by most or all of the molecules of interest. Genes that appear to be upregulated in both cell lines by all the ingredients include: ACLY, AQP3, COX1, NOS3, and PLOD3. Genes that appear to be downregulated in both cell lines by all ingredients include only PGR.

## 1. Introduction

A *PubMed* (http://www.ncbi.nlm.nih.gov/sites/entrez?cmd=&db=pubmed) search using the following keywords, “Antioxidant + Skin”, will return approximately 7786 individual journal hits. The earliest references are papers examining Vitamin E in dermatology and Ascorbic Acid use in skin tuberculosis dated to 1950. Expanding the search to the following keywords “Antioxidants + Skin + Review” provides approximately 878 individual journal hits. These searches demonstrate very clearly that the field of antioxidant research related to skin is replete with individual contributions examining antioxidant activity and skin biology including, for example, the role of plant extracts, vitamins, and minerals in skin antioxidant applications. 

There have been some very good fundamental references on the role of antioxidants in human health and in particular as they apply to the skin. Important references are those edited by Mukhtar [1], Packer and Cadenas [2] and Packer and Valacchi [3]. While it would be nearly impossible to summarize all the pertinent references on antioxidants and their role in skin pharmacology and physiology, several recent references of interest include papers by Cooke and Evans [4], Nicholas and Katiyar [5], Epstein [6], Ditre et al. [7], Allemann and Baumann [8] and Arct and Pytkowska [9]. 

However, cutaneous research examining the influence of antioxidants from a genomic perspective is presently lacking. There have been some studies examining the global genomic effects of antioxidants on dietary health [10]. In addition, some studies report examining unique cells line, in particular cancer cell lines, as they respond to antioxidant treatments using human genomic test methods [11]. A paper recently published examined the effects of UV light on dorsal root ganglion and the subsequent effects of the nutrient media from the irradiated nerve cells on NHEK using human microarrays [12]. 

In the present work, normal human dermal fibroblasts (NHDFs) and normal human epidermal keratinocytes (NHEKs) were treated with various well-known antioxidants including Resveratrol [13], Epigallocatechin Gallate (EGCG) [14], Genistein [15], Rosavin [16], Puerarin [17], Chlorogenic Acid [18], Propolis [19], and two newer unexplored isoflavonoids isolated from *Maclura pomifera *(Osage Orange) which include Pomiferin and Osajin [20]. Treatment of the individual cell lines for 24 hours with purified samples of the antioxidants was followed by examination of the treated cells using human microarray analysis. A working hypothesis which guided this study was that there may be common genomic influences which all antioxidants have on skin cells that are the more critical genes that provide the beneficial effects of the antioxidants.

## 2. Materials and Methods

**Figure 1:**
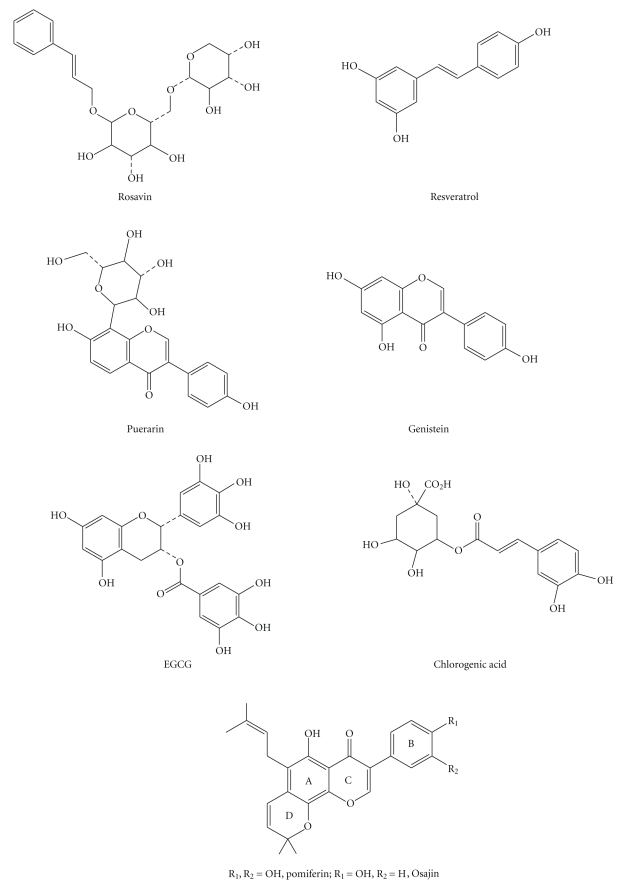
Chemical structure of antioxidant ingredients tested.

The individual ingredients used in this study were examined for their cytotoxicity on Normal Human Dermal Fibroblasts and Normal Human Epidermal Keratinocytes using the standard MTT assay. Where possible, the samples were tested at similar concentrations of 0.01% unless the ingredients proved to be either cytotoxic or well tolerated at a higher dose. In which case, they were tested at the highest nonlethal dose possible. Two exceptions were Puerarin and Propolis which were tested at 0.1% in these studies. The chemical structures of all the ingredients except Propolis are shown below. Propolis was exceptional in this study as it is a composition extracted from Honeycomb which is a complex combination of polyphenols, isoflavonoids, and flavonoids. The Propolis used in this study was supplied by Lisoma and is suggested to be 80% pure in propolis content. Resveratrol (99%), Genistein (>95%), and Chlorogenic Acid (>95%), were purchased from Sigma Chemical Company and were used without further purification. Purified Rosavin (96%), EGCG (97%), and Puerarin (96%), were provided by Chromadex Chemical Company. Pomiferin (95%) and Osajin (90%) were provided by Gaia Chemical. All chemical purities were verified by HPLC analysis. Chemical structures for the various test materials are shown in the compiled Figure 1. The concentrations of the actives tested on NHEK and NHDF are shown in Table 1. 

Human epidermal keratinocytes and dermal fibroblasts were obtained from Cascade Biologics. Keratinocytes were grown in Epilife media (supplemented per the manufacturer's recommendation) while fibroblasts were grown in DMEM (supplemented with 1.5% fetal bovine serum for the array treatments). Both cell types were seeded into T-25 flasks and grown at 37 ± 2°C and 5 ± 1% CO_2_ until confluent. Upon reaching confluency the cells were treated with the various antioxidants (dissolved in DMSO if needed, with a final DMSO concentration in the media of 1%) for 24 hours.

After the 24 hour treatment, total RNA was isolated using an RNAqueous Kit (Ambion) per the manufacturer's instructions. After purification, the total RNA was prepared for array use by first amplifying the RNA using a MessageAmp aRNA Kit (Ambion), and then fluorescently labeling the aRNA with Cy3 or Cy5 using an ASAP Labeling Kit (Perkin Elmer), both per the manufacturer's instructions. To purify the fluorescently labeled aRNA, a microcon YM-30 filter column was inserted into a collection tube and filled with 400 *μ*l of TE buffer. The Cy3 and Cy5 probes were combined and then added to the microcon filter and thoroughly mixed with the TE buffer. The filter was centrifuged at 12,000 RPM for 8 minutes and the flow through was discarded. The column was then washed twice with 400 *μ*l of TE buffer, discarding the flow though each time. After the final wash the filter column was inverted, placed into a new collection tube and centrifuged at 2,000 RPM for 2 minutes to collect the probe. 

The fluorescently labeled aRNA was applied to the DNA microarray chips (Agilent Technologies) and the chip was hybridized overnight and washed per the manufacturer's recommended protocol. After washing, the microarrays were scanned with an Axon GenePix 4100A Scanner with the scanning resolution set to 5 *μ*m and analyzed with GenePix Pro software. During the initial scan the PMT gains for the scanner were adjusted such that the cy5/cy3 image count ratios are between 0.88 and 1.12.

Fluorescence intensities for the microarrays were subjected to global normalization. The total fluorescent signal for both dyes was normalized with a correction factor that would make the ratio of total intensities for both dyes equal to one. For this study a Cy3/Cy5 (untreated/treated) fluorescence intensity ratio greater than 1.3 or less than 0.7 (this relates to a change in gene expression of at least ±30%) was used as the cutoff for up- and downregulated genes, respectively. This is referred to as the “ratio of medians” in the array summaries. This cutoff ratio falls within the typical range of cutoff ratios found in the literature [21]. In addition, the fluorescence intensity of the gene marker had to be greater than the background intensity.

## 3. Results and Discussion

 A summary of the genes examined in these studies is shown in Table 2. Selection of the genes was principally the effort of the authors to narrow the extensive list of genes found in each array to some key target genes felt to be critical for skin. Genes shown in the Table 1 are not grouped in any particular order; they are shown alphabetically as they appear in the arrays. 

**Figure 2:**
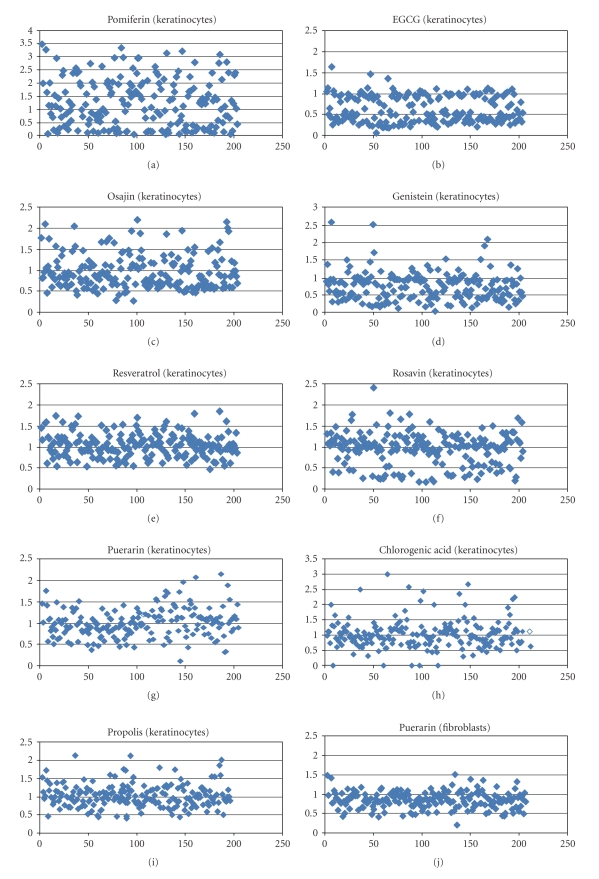

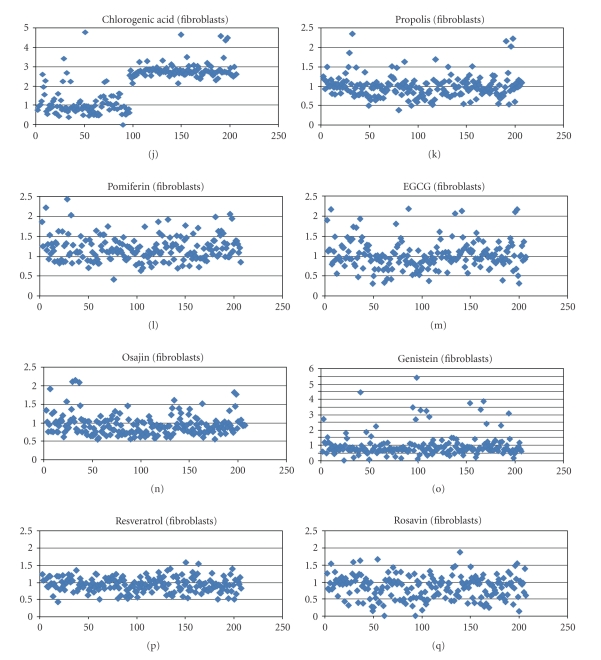
Ratio of Medians for genes shown in Table 2 for all individual product treatments on NHEK and NHDF.

Provided in Figure 2 are summary graphs showing the Ratio of Median (ROM), *vide supra*, for the compiled gene data examined for the various ingredients tested at the concentrations specified in the Methods section on either NHEK or NHDF as indicated. 

Selection of genes of interest was done to cover a broad range of skin functions including, for example, extracellular matrix protein expression, lipid synthesis, energy and metabolism, intrinsic antioxidant synthesis, ROS and DNA repair response, skin polysaccharide and glycoprotein synthesis, hormone response, longevity, cellular differentiation, nerve growth and protection, retinol response, circadian influences and skin pigmentation. 

Determination of which genes appear to be regularly up- or downregulated by the majority of the antioxidants examined was done by comparing the data across each gene and test material looking for genes that showed Ratio of Medians greater than 1.3 or less than 0.7 consistently. If more than four of the entries showed data suggesting either upregulation or downregulation, the gene set was included. The summary of the genes that were noted to be either upregulated or downregulated is provided in Tables 3, 4, 5, and 6. It seems that the keratinocytes respond more broadly to the antioxidant treatments than the fibroblasts as noted by the greater preponderance of gene responses, both up and down, for the keratinocytes verses the fibroblasts. 

In reviewing the data compiled in Tables 3–6, it is possible to narrow the genes that are “universally” upregulated or downregulated for each cell line. It is noted that the following genes are upregulated in both fibroblasts and keratinocytes: ACLY, AQP3, COX1, NOS3, and PLOD3. Interestingly, only one gene is commonly downregulated in both cell lines by the majority of the antioxidant treatments: PGR. 

 Demonstrating that the Progesterone Receptor (PGR) gene would be downregulated by the majority of the ingredients examined in these studies in both cell lines suggests that this particular gene and its corresponding protein may play a more pivotal role in the behavior of these ingredients on skin than previously realized. It has been shown that progesterone applied to the skin of monkeys suppresses estrogen receptor expression [22–24]. In reviewing the effect of these ingredients on the estrogen receptor gene expression (Table 7) there appears to be little indication that these molecules stimulate expression of the two key estrogen receptor genes. In most cases, the ingredients had no effect on the estrogen receptor gene expressions (indicated by Ratio of Medians between 0.7 and 1.3). This suggests that while it is well known that some molecules such as genistein can bind to the estrogen receptors it seems unlikely that all of these molecules would do so. What may be happening with many (or all) of these unique ingredients on skin could be an altering of the estrogen/progesterone receptor protein ratio for each cell line. If these molecules universally suppress expression of the progesterone receptor proteins while not influencing the expression of the estrogen receptor proteins, this may cause a change in the critical ratio of these two key proteins. In this case, what appears as an estrogenic effect is, instead, a suppression of progesterone effects due to a diminishment in expression of the receptor proteins. This suggests that the term “phytoestrogen” often used to describe molecules like genistein may be somewhat of a misnomer and the bulk of these topically beneficial molecules are actually “anti-phytoprogesterones”. Most certainly, the majority of the ingredients examined in this study appear to be progesterone receptor gene antagonists although confirmation of these influences via protein assays remains to be done.

 Valacchi et al. have examined the role of antioxidants in skin stressed with the oxidizer ozone, looking at the antioxidant  *β*-Carotene in particular. These authors looked at inflammatory markers such as iNOS [NOS1] and Hemeoxygenase-1 [HO1] when skin is stressed with ozone and the role that *β*-Carotene plays in minimizing inflammatory response [25]. They noted that in the presence of an external oxidative stress such as ozone, antioxidants such as  *β*-Carotene will generally downregulate inflammatory markers such as those noted above. However, without the presence of an external oxidative threat, it was found in the current studies that in both cell lines, direct application of the antioxidants used in these studies under ambient conditions resulted in upregulation of the inflammatory mediators COX1 and NOS3 [26–29]. This is somewhat surprising as typically commercial descriptions of antioxidants often suggest they are soothing or calming. It would seem that just the opposite may be occurring and the majority of the various antioxidants tested here cause inflammatory-like responses in the skin cells. However, the close balance the body and skin maintain between healing and inflammation may suggest that there is a common influence of the majority of these antioxidants to stimulate a healing type response in the skin cells. There appears to be an inverse relationship between COX1 expression and Progesterone expression, so the possibility that the PGR gene is downregulated by ingredients that also appear to upregulate COX1 may be expected [30]. 

The common upregulation of ATP-Citrate Lyase (ACLY) in both cell lines by the majority of the antioxidants is also quite interesting [31, 32]. ATP-Citrate Lyase is a critical enzyme responsible of *de novo* fatty acid synthesis responsible for generating cystolic acetyl-CoA and oxaloacetate. Acetyl-CoA is one of the essential building blocks for lipid synthesis in the body. Common upregulation of this gene suggests that the majority of the antioxidants tested here may be influencing skin lipid synthesis. This would support the beneficial effects of the ingredients on the skin as well, particularly for barrier repair and improvements. 

Aquaporin-3 has only recently emerged as a critical protein in skin [33, 34]. The protein is known to be expressed in both keratinocytes and fibroblasts. The protein controls water flux within the skin cells. In fibroblasts and keratinocytes, the Aquaporin-3 protein has been suggested to be involved in wound healing through cell migration processes [33]. Its common upregulation by the antioxidants in this study suggests a key role for this protein in improved skin health and cell turnover as well as playing an important role in skin hydration.

Lysine hydroxylase-3 (PLOD3) is the gene responsible for expression of a protein that helps crosslink collagen and elastin fibers via hydroxylation of lysine and proline residues [35]. Its expression in fibroblasts is well established, however, it is unclear if it is expressed in keratinocytes or not. It can be noted that topical application of the majority of the isoflavonoids tested causes upregulation of PLOD3 in both fibroblasts and keratinocytes. Certainly, upregulation of PLOD3 would be consistent with the finding that topical applications of isoflavonoids lead to firmer looking skin as increased crosslinking of collagen would lead to a rebuilding effect in aging skin. 

While it is desirable to think that the bulk of the molecules tested here work through some uniform mechanistic pathways, this idea may be somewhat naive. However, by examining multiple structurally unique molecules well-known to influence skin structure and function, a clearer picture of the genomic effects of these ingredients on skin may begin to be elucidated. Certain targets, in particular, the unusual finding that the bulk of these ingredients appear to downregulate Progesterone Receptor gene expression, warrant further protein work to verify if this is indeed the case. It may be that while unique in chemical structure, common pathways to improved skin appearance are not as unique to the molecules tested, but rather to the targets that they address universally. The ability to examine multiple gene expression effects using human microarrays will open future doors to answer these types of questions.

## Figures and Tables

**Table 1:** Concentrations of various ingredients tested on Keratinocytes and Fibroblasts.

Ingredient	Keratinocytes	Fibroblasts
Pomiferin	0.01%	0.01%
Osajin	0.0001%	0.0001%
Puerarin	0.1%	0.1%
Rosavin	0.01%	0.01%
Chlorogenic Acid	0.001%	0.001%
EGCG	0.01%	0.01%
Genistein	0.01%	0.01%
Resveratrol	0.05%	0.05%
Propolis	0.10%	0.10%

**Table 2:** Summary of Genes Examined.

Gene Symbol	Gene Symbol	Gene Symbol	Gene Symbol	Gene Symbol
ACLY	DHCR24	KLK7	POMC	USF1
AGER	DHCR7	KRT1	PPARA	UVRAG
AGK	DPT	KRT5	PPARD	VDR
AIFM1	DSG3	KRT15	PPARG	VEGFA
AQP1	EGF	LAD1	PRDX1	XPA
AQP3	EGFR	LAMA3	PRDX2	XPC
AR	ELN	LAMB1	PRDX5	
ARPC1A	ENDOG	LAMC1	PTDSS1	
ARPC3	ESR1	LIAS	PTGER1	
ATM	ESR2	LIG1	RAD23A	
ATR	FAP	LOR	RAD23B	
BCDO2	FBN1	LOX	RARA	
BCL2	FBN2	LSS	RARB	
BCL2	FGF1	MC1R	RARG	
BCL2A1	FGF2	MCHR1	RBP1	
BCL2L1	FGFR1	MLPH	RXRA	
BECN1	FLG	MMP1	RXRB	
CASP7	FLNA	MMP2	SCEL	
CASP14	FN1	MOAP1	SIRT1	
CAT	FOXO3A	MTCH1	SIRT2	
CCS	FSHR	MTCO1	SIRT3	
CD44	GAPDH	MTCO2	SIRT4	
CDH1	GLRX2	MTNR1A	SIRT5	
CH25H	GPX1	MVD	SIRT6	
CIRBP	GPX4	MVK	SIRT7	
CLOCK	GPX5	NAGK	SOD1	
COL1A1	GRN	NFKB	SOD2	
COL1A2	GSR	NGB	SOD3	
COL4A1	HAS1	NGFB	SPON1	
COL4A2	HAS2	NGFRAP1	SQLE	
COL6A1	HIF1A	NOS1	SRD5A1	
COL7A1	HIF3A	NOS2	SRD5A2	
COL17A1	HMGCR	NOS3	TDG	
COQ10A	HSPA1A	NOSTRIN	TERT	
COX1	HSPA5	OA1	TGFA	
CRABP1	HSPB1	OGG1	TGFB1	
CRABP2	HYAL1	OXR1	TGM1	
CYGB	HYAL2	OXSR1	TGM3	
CYP46A1	IGF1	P4HA1	TIMELESS	
CYP51A1	IGF2	P4HA2	TIMP1	
DDB1	IGF1R	P4HB	TIMP2	
DDB2	IL1A	PAK2	TIMP4	
DDIT3	IL1B	PER1	TNF	
DDIT4	ITGA1	PER2	TP53	
DDI2	ITGA5	PGM1	TPT1	
DDT	ITGA6	PGR	TRPV1	
DEFA3	ITGA7	PLOD	TXN	
DEFB1	IVL	PLOD2	TYR	
DEFB104	KL	PLOD3	TYRP1	
DEFB4	KLK5	PNN	UNG	

**Table 3:** Summary of fibroblasts genes noted to be commonly upregulated by the antioxidant treatments.

FIBROBLASTS	Test Materials	Pomiferin	Osajin	Resveratrol	EGCG	Genistein	Rosavin	Puerarin	Chlorogenic Acid	Propolis
Gene Name	Gene Description									

ACLY	ATP citrate lyase	1.874	1.334	1.229	1.896	0.582	1.248	1.504	0.766	1.254
AQP1	Aquaporin 1	2.232	1.915	1.093	2.166	0.472	1.531	1.436	1.231	1.499
COL1A1	Collagen, Type 1, alpha 1	2.446	1.36	1.074	1.423	0.757	1.237	1.017	2.26	1.496
COX1	Cytochrome c oxidase 1	1.875	2.089	1.288	1.929	0.699	1.623	1.178	2.252	1.485
GRN	Granulin	1.394	1.25	1.233	1.444	0.753	1.288	1.13	1.502	1.478
NOS3	Nitric oxide synthase 3	1.478	1.607	1.39	2.061	0.717	1.459	1.527	0.874	1.302
PLOD3	Lysine hydroxylase 3	1.782	1.367	1.573	1.16	0.687	1.442	1.404	3.805	1.186
RARA	Retinoic acid receptor, alpha	1.72	1.513	1.534	1.36	0.706	1.211	1.379	1.423	1.034
TXN	Thioredoxin	1.956	1.767	1.387	2.164	0.692	1.538	1.341	3.556	2.229

**Table 4:** Summary of fibroblasts genes noted to be commonly downregulated by the antioxidant treatments.

FIBROBLASTS	Test Materials	Pomiferin	Osajin	Resveratrol	EGCG	Genistein	Rosavin	Puerarin	Chlorogenic Acid	Propolis
Gene Name	Gene Description									

DSG3	Desmoglein 3	0.847	0.545	0.708	0.667	−0.118	0.385	0.438	0.46	0.737
HAS1	Hyaluronan synthase 1	1.091	0.667	0.526	0.615	0.695	0.429	0.462	1.5	0.385
ILIA	Interleukin 1, alpha	1.2	0.599	0.588	0.556	−0.27	0	0.5	0.517	0.5
KL	Klotho	1.136	0.731	0.56	0.7	0.099	0.167	0.588	0	0.7
NOS2	Nitric oxide synthase 2	0.831	0.694	0.655	0.928	0.303	0.441	0.588	1.05	0.724
PGR	Progestone receptor	0.764	0.926	0.579	0.571	0.337	0.364	0.583	0	0.545

**Table 5:** Summary of keratinocyte genes noted to be commonly upregulated by the antioxidant treatments.

KERATINOCYTES	Test Materials	Pomiferin	Osajtn	Resveratrol	EGCG	Genistein	Rosavin	Puerarin	Chlorogenic Acid	Propolis
Gene Name	Gene Description									

ACLY	ATP citrate lyase	3.499	1.769	1.46	1.047	0.887	1.319	1.461	0.981	1.541
AQP1	Aquaporin 1	3.288	2.098	1.575	0.436	0.947	1.34	1.757	2	1.736
AQP3	Aquaporin 3	1.65	1.055	1.598	1.636	2.587	1.17	1.418	1.319	1.451
CD44	Transmembrane glycoprotein CD44	2.312	1.345	1.236	0.867	1.504	0.758	1.07	1.34	1.398
CDH1	Cadherin 1	2.489	1.489	1.599	0.989	1.152	0.934	1.379	1.582	1.422
COX1	Cytochrome c oxidase 1	2.637	2.045	1.421	0.985	0.914	1.354	1.217	2.499	2.149
FGFl	Fibroblast growth factor 1	2.65	1.67	1.519	0.194	0.316	1.303	1.345	3	0.945
GRN	Granulin	2.976	1.652	1.496	0.959	0.936	1.281	1.297	1.452	1.57
HSPB1	Heat Shock 27 kD protein 1	2.991	1.437	1.539	1.028	0.922	1.406	1.42	2.581	1.768
KRT5	Keratin 5	2.964	2.197	1.704	1.008	0.573	1.258	1.134	2.439	2.141
NOS3	Nitric oxide synthase 3	3.152	1.859	1.609	0.981	0.893	1.277	1.311	1.041	1.812
PLOD3	Lysine hydroxylase 3	3.231	1.94	1.45	1.102	0.917	1.299	1.481	2.671	1.756
TPTl	Histamine-releasing factor	2.813	2.148	1.617	1.105	0.925	1.363	1.431	2.184	1.874
TXN	Thioredoxin	2.41	1.924	1.369	0.985	0.83	1.352	1.206	2.244	2.026

**Table 6:** Summary of keratinocyte genes noted to be commonly downregulated by the antioxidant treatments.

KERATINOCYTES	Test Materials	Pomiferin	Osajin	Resveratrol	EGCG	Genistein	Rosavin	Puerarin	Chlorogenic Acid	Propolis
Gene Name	Gene Description									

AR	Androgen receptor	0.06	0.462	0.607	0.257	0.302	0.417	0.581	0	0.459
CYGB	Cytoglobin	0.175	0.414	0.636	0.319	0.242	0.268	0.571	0.688	0.667
EGF	Epidermal growth factor	0.159	0.52	0.741	0.25	0.204	0.272	0.516	0.471	0.774
ESR2	Estrogen receptor 2	0.093	0.444	0.706	0.192	0.727	0.26	0.478	0	0.438
FBN1	Fibrillin 1	0.52	0.78	0.75	0.215	0.214	0.337	0.667	0.563	0.612
FBN2	Fibrillin 2	0.731	0.917	0.857	0.32	0.473	0.541	0.693	0.571	0.634
IGF1	Insulin growth factor 1	0.135	0.588	0.6	0.31	0.429	0.381	0.5	0	0.462
KL	Klotho	0.038	0.273	0.545	0.357	0.278	0.181	0.444	0	0.4
MC1R	Melanocortin 1 receptor	0.167	0.571	0.619	0.302	0.433	0.19	0.561	2	0.538
PGR	Progestone receptor	0.052	0.538	0.8	0.304	0.217	0.293	0.235	2	0.5
POMC	Proopiomelanocortin	0.194	0.483	0.75	0.295	0.452	0.357	0.788	0.982	0.738
PPARG	Peroxisome proliferator activated receptor, gamma	0.154	0.563	0.571	0.314	0.129	0.538	0.45	0.333	0.438
PTGER1	Prostaglandin E receptor 1	0.107	0.6	0.667	0.209	0.235	0.245	0.657	1.25	0.629
RAD23A	RAD23 homolog A	0.391	0.482	1.034	0.385	0.414	0.578	0.971	0.577	0.789
RXRA	Retinoid X receptor, alpha		0.636		0.445	0.409	0.561		0.621	0.689
SOD3	Superoxide dismutase 3	0.13	0.571	0.467	0.276	0.431	0.617	0.6	0.571	0.778
SRD5A2	Steroid 5 alpha-reductase A2	0.097	0.556	0.636	0.371	0.333	0.588	0.529	0.5	0.583
TERT	Telomerase reverse transcriptase	0.311	0.595	0.619	0.298	0.394	0.436	0.556	0.709	0.756
TYRP1	Tyrosinase related peptide 1	0.043	0.852	0.611	0.34	0.244	0.296	0.7	0.5	0.5

**Table 7:** Summary of Ratio of Medians for ingredient influences on estrogen receptor gene expression.

	Pomiferin (ROM)	Osajin	Resveratrol	EGCG	Genistein	Rosavin	Puerarin	Chlorogenic Acid	Propolis
Keratinocyte Gene									
ESR1 (Estrogen Receptor 1)	0.538	0.824	1.111	0.205	0.22	0.744	0.988	0.714	0.795
ESR2 (Estrogen Receptor 2)	0.093	0.444	0.706	0.192	0.727	0.26	0.478	0	0.438

Fibroblast Gene									
ESR1 (Estrogen Receptor 1)	0.824	0.728	0.784	0.65	0.664	0.294	0.708	0.5	1
ESR2 (Estrogen Receptor 2)	0.828	0.864	0.5	0.333	0.984	0	0.545	1	0.7
